# Local habitat conditions explain the variation in the strength of self-thinning in a stream salmonid

**DOI:** 10.1002/ece3.1591

**Published:** 2015-07-15

**Authors:** Knut Marius Myrvold, Brian P Kennedy

**Affiliations:** 1 Department of Fish and Wildlife Sciences, University of IdahoMoscow, Idaho, 83844-1136, Unites States; 2 Departments of Biological Sciences and Geological Sciences, University of IdahoMoscow, Idaho, 83844-1136, Unites States

**Keywords:** Bioenergetics, density dependence, Idaho, *Oncorhynchus mykiss*, regulation, steelhead

## Abstract

Self-thinning patterns are frequently used to describe density dependence in populations on timescales shorter than the organism's life span and have been used to infer carrying capacity of the environment. Among mobile animals, this concept has been used to document density dependence in stream salmonids, which compete over access to food and space. The carrying capacity, growth conditions, and initial cohort sizes often vary between streams and stream sections, which would influence the onset and strength of the density dependence. Despite much effort in describing habitat relationships in stream fishes, few studies have explicitly tested how the physical environment affects the slope of the thinning curves. Here, we investigate the prevalence and strength of self-thinning in juvenile stages of a steelhead (*Oncorhynchus mykiss*) population in Idaho, USA. Further, we investigate the roles of local physical habitat and metabolic constraints in explaining the variation in thinning curves among study sites in the watershed. Only yearling steelhead exhibited an overall significant thinning trend, but the slope of the mass–density relationship (−0.53) was shallower than predicted by theory and reported from empirical studies. There was no detectable relationship in subyearling steelhead. Certain abiotic factors explained a relatively large portion of the variation in the strength of the self-thinning among the study reaches. For subyearling steelhead, the slopes were negatively associated with the average water depth and flow velocity in the study sites, whereas slopes in yearlings were steeper in sites that incurred a higher metabolic cost. Our results show that the prevalence and strength of density dependence in natural fish populations can vary across heterogeneous watersheds and can be more pronounced during certain stages of a species' life history, and that environmental factors can mediate the extent to which density dependence is manifested in predictable ways.

## Introduction

One of the primary interests in ecology is identifying the patterns and processes that govern population growth. Population size and growth rate can be *limited* by factors such as weather that act independently of population size, and *regulated* by density-dependent factors, when the per capita growth rate of the population depends on its own density (Sinclair and Pech 1996; Sibly and Hone 2002). Which of the two processes has a stronger effect on populations has been heavily debated (Nicholson 1933; Andrewartha and Birch 1954), but there is general consensus that most populations are governed by a combination, rather than one or the other (Leirs et al. 1997; Karels and Boonstra 2000). Their relative importance varies by population, depending upon abiotic conditions, community organization, and the population's size and trajectory (Begon et al. 1996; Einum 2005).

Detecting density dependence in populations over time intervals shorter than a single generation length has received much attention in agronomy and applied ecology (Westoby 1984). One particularly interesting pattern was formalized by researchers observing how tree mortality was proportional to the average mass of trees in the stand, as the stand was growing (a pattern known as *self-thinning*; Yoda et al. 1963). Animal populations also thin over time, but there is rarely a factor that governs mobile animal taxa such as the shared need for light among plants (Begon et al. 1986, 1996). Among mobile animals, the self-thinning framework has been invoked to describe numerical changes in stream salmonids as cohorts are maturing (Grant and Kramer 1990; Grant 1993, Bohlin et al. 1994), and has later been applied to other stream salmonid systems (e.g., Elliott 1993; Dunham and Vinyard 1997; Rincón and Lobón-Cerviá 2002; Keeley 2003). A relatively confined range, high reproductive capacity, and limited dispersal during summer have made salmonids a tractable system for the study of this phenomenon. The processes creating self-thinning patterns have been attributed to competition over food and space, although we note that other factors than competition alone can affect the population size as individuals grow in size, causing apparent thinning patterns. The mass–density relationships follow power functions due to nonlinear energy conversion with size (Bohlin et al. 1994; Steingrimsson and Grant 1999) and behavioral mechanisms regulating territory size (Grant et al. 1989; Grant and Kramer 1990) (*see* Keeley 2003 for a review of these hypotheses).

Although the territory size hypothesis has been implicitly tested in many studies, habitat factors have seldom been explicitly considered in studies on self-thinning, with a few notable exceptions (Steingrimsson and Grant 1999; Lobón-Cerviá 2008). As Lobón-Cerviá (2008) noted, most studies have assumed no effect from habitat factors on the self-thinning patterns in salmonids (e.g., Elliott 1993; Grant and Imre 2005; Imre et al. 2005), which would be unlikely in streams experiencing seasonal flooding and drought. Further, habitat quality, thermal regime, proximity to spawning grounds, and productivity can vary greatly within a watershed, all affecting initial densities, survival, and growth opportunities for juvenile salmonids (Gibson 2002; Ebersole et al. 2006; Myrvold and Kennedy 2015a). This variation could therefore manifest in varying degrees of thinning throughout the stream network as cohorts experience different conditions depending on their location.

**Figure 1:**
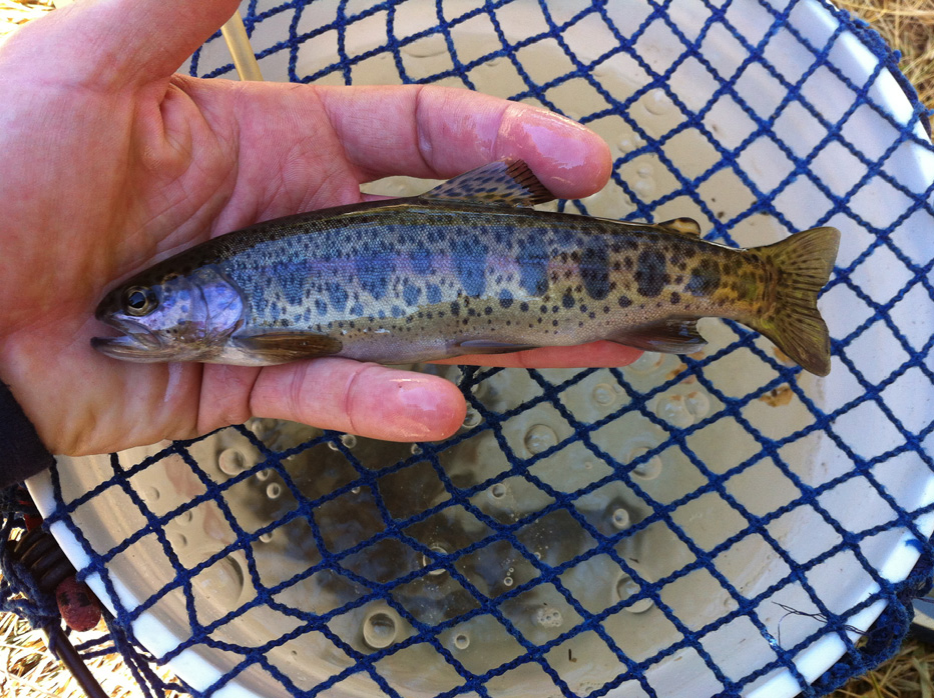
A yearling steelhead (*Oncorhynchus mykiss*) in Lapwai Creek, Idaho, USA.

Here, we test the hypothesis that cohorts of juvenile steelhead (Fig. 1) will thin according to predictable relationships (Grant and Kramer 1990; Bohlin et al. 1994; Steingrimsson and Grant 1999) in a watershed in Idaho, USA, designated as critical habitat for a population of steelhead that is listed as threatened under the Endangered Species Act. Following Steingrimsson and Grant (1999) and Lobón-Cerviá (2008), we wanted to understand how local environmental factors could influence the thinning slopes. We first analyze the extent to which juvenile steelhead cohorts across the watershed self-thin and second how the variation in the thinning slopes among study sites can be attributed to local physical habitat conditions and bioenergetic constraints. The factors assessed are known to influence local densities and individual growth opportunities (Bjornn and Reiser 1991; Quinn 2005; Myrvold and Kennedy 2015a,b). Because initial densities vary, the carrying capacity likely varies across time and space, and steelhead have different habitat requirements throughout ontogeny, we expect that the slopes of the overall thinning curves will be shallower than predicted by the food consumption (−0.73), metabolic rate (−0.87), and space hypotheses (−0.86) (Grant and Kramer 1990; Bohlin et al. 1994, Steingrimsson and Grant 1999). We expect that there will be differences in slopes among the study sites and that this variation can be explained by habitat factors known to influence steelhead growth and density.

## Materials and Methods

### Study area and population

**Figure 2:**
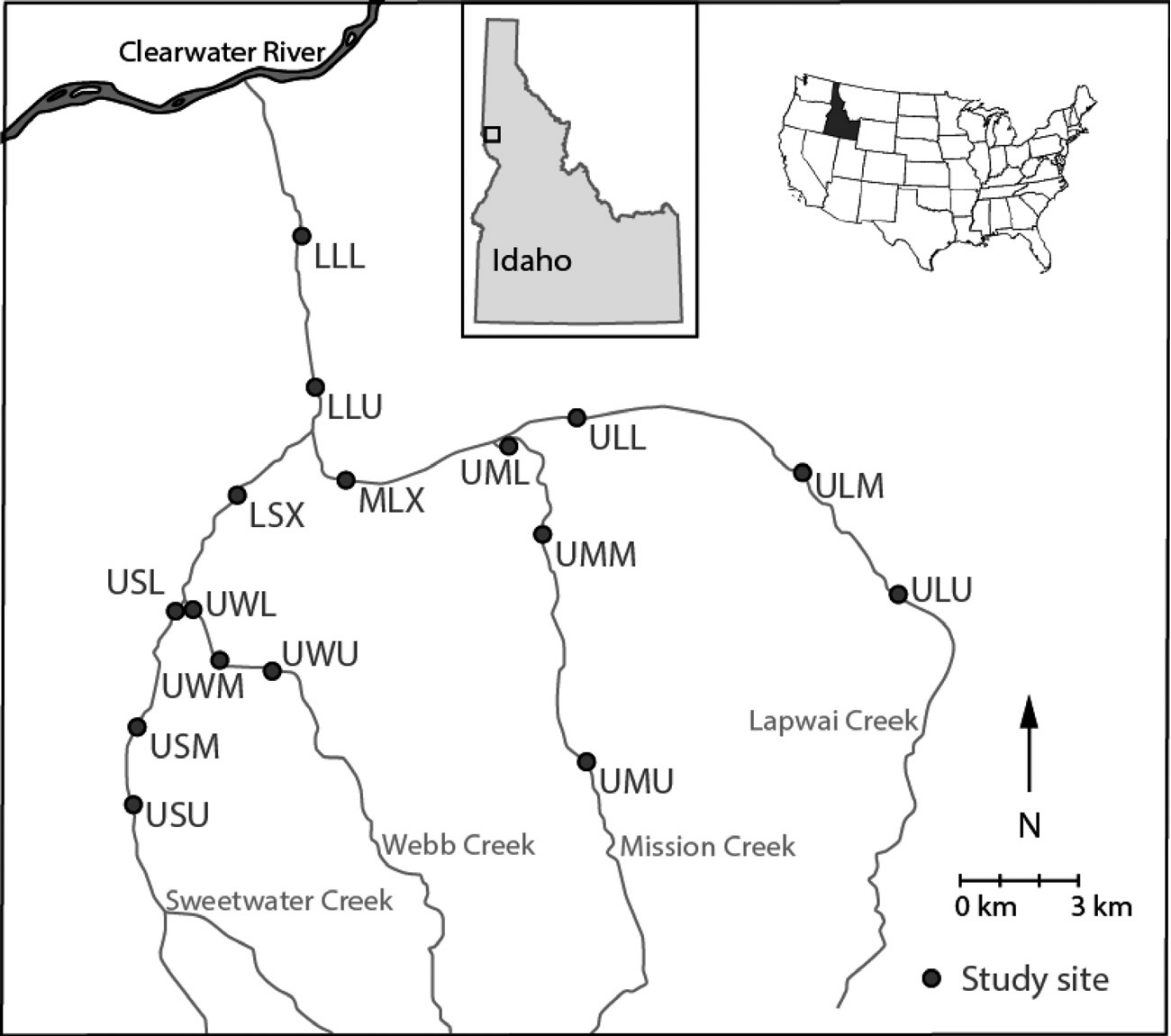
The map shows the four major streams of the Lapwai Creek watershed and its location in North Central Idaho, USA (insert). The watershed is part of the Columbia River Basin which drains to the Pacific Ocean. The study sites were sampled on average five times in 2010 and five times in 2011.

The study was conducted in the Lapwai Creek watershed in Idaho, USA (Fig. 2). Details on the study area, design, and sampling methods have been reported elsewhere (Myrvold and Kennedy 2015a), but we include a description in the following. There are four major tributaries in the 694-km^2^ watershed that together form the fourth-order Lapwai Creek, which empties into the Clearwater River (elevation 237 m). The streams drain the north slopes of Craig Mountain (elevation 1530 m) and carve steep canyons through the landscape. Mean annual precipitation is 490 mm, with larger amounts falling at higher elevations, primarily from October through May. The plateau above the escarpment is overlain with loess, and the predominant land use is dryland grain agriculture, which covers 34% of the watershed. Coniferous forests cover 29%, primarily at higher elevations above the prairie, and grasslands dominate the steep canyon sides and valley floors (Homer et al. 2007).

Snake River steelhead and salmon (*Oncorhynchus* sp.) have declined in numbers since the 1870s due to hydropower development, overharvest, ocean conditions, and habitat degradation (Chapman 1986; NRC 1996). Although habitat quality in lower sections of the Clearwater River has been significantly affected by land use alterations, particularly in low-elevation arable areas, records suggest that streams in the Lapwai Creek watershed produced significant numbers of anadromous fish in the past (Johnson and Stangl 2000; NMFS 2006). In recent years, juvenile coho salmon smolts (*Oncorhynchus kisutch*) have been stocked as part of a reintroduction program in lower reaches of the system, but individuals are generally not sympatric in space or time with steelhead. No hatchery supplementation exists for steelhead in the watershed.

### Study design

Our study was motivated by identifying the variation in the strength of density dependence and its potential mediators in a stream system that is characterized by variable habitat conditions. To span a gradient of physiographic and land-use conditions, we therefore randomly assigned three study reaches on each of the tributaries, one below the confluence of each pair and two study reaches on the main stem, totaling 16 sites (Myrvold and Kennedy 2015a). Each of the 16 sites was sampled on average 5 times between mid-June and early November of both 2010 and 2011. From this, we could measure the size distribution of steelhead cohorts and estimate population size, as well as quantify the physical habitat characteristics and thermal regime. Because energy expenditure in fishes increases nonlinearly with temperature, we calculated the energetic cost incurred by the local temperature regimes as described below.

### Sampling methods and material

#### Steelhead data

We began fish sampling when the flows were low enough to permit efficient electrofishing. Block nets were set to ensure a closed population, and three-pass depletion electrofishing was conducted using a Smith-Root LR-24 backpack electroshocker (Smith-Root Inc., Vancouver, WA). To reduce the stress of capture and handling on fish, sampling was conducted during early morning hours when the water temperatures did not exceed 18°C. We measured fork length in millimeters and weight to the nearest decigram. Upon completion of sampling, we removed the block nets and released fish back to the study reach. Based on the size frequency distribution, individuals were characterized as *subyearling* (hatched earlier in the calendar year) or *yearling* (hatched in a previous year). Densities of each age class were calculated using Carle and Strub's (1978) maximum weighted likelihood estimator of multiple-pass removal data. Due to the small size and low discharge of these streams, we obtained high capture probabilities (season averages ±SD were 0.63 ± 0.14 in 2010 and 0.62 ± 0.13 in 2011, respectively) and consequently narrow confidence intervals around our population estimates. We only included sampling events when the respective age classes were present and effectively captured. Steelhead smaller than 40-mm fork length did not recruit to our sampling gear, which was often the case on the first visit each season. Consequently, when the majority of the subyearlings were not captured effectively, density estimates were unobtainable and not included in our analysis. All fish sampling and handling procedures were permitted as part of the section 7 consultation for the Lewiston Orchards Biological Opinion (NMFS 2006, 2010), Idaho Department of Fish and Game, and the University of Idaho Institutional Animal Care and Use Committee.

#### Habitat data

To quantify habitat variables that have been shown important to juvenile salmonids (Bjornn and Reiser 1991; Quinn 2005; Chun et al. 2011), we established transects perpendicular to the channel at 5-m intervals throughout each study site. The channel was first characterized into predominant geomorphic unit at each transect (riffle, run, pool, or glide), and percentages of each were calculated to express the channel's composition. *Riffle* refers to fast-flowing, shallow, and turbulent water with visible waves on the water surface; *run* refers to fast, but less turbulent, flow in deeper water; and *glide* refers to shallow, gentle-flowing current (Fisher et al. 2012). Transects were split into 5 sections of equal width. At each point, we measured the velocity (ms^−1^; Marsh McBirney, Loveland, CO), depth (cm), and the longest axis of a haphazardly selected piece of substrate (mm). The approach yielded approximately 100 point measurements of each of the physical variables, from which we calculated the average values (Table 1). We also calculated the percentage of the reach being composed of shallow and slow-flowing water (<6 cm deep with flow velocity <0.15 ms^−1^, denoted *slow and shallow* in the tables), as these features are important for subyearling steelhead (Bjornn and Reiser 1991).

**Table 1:** Sample characteristics of the 16 study sites in the Lapwai Creek watershed, USA, showing the average mass of subyearling and yearling steelhead in August 2011, the elevation of the site and the channel width, and the average channel covariates and their standard deviations (in parentheses) used in the modeling of this study. *Slow and shallow* refers to the proportion of the study reach that was <6 cm deep with flow velocity <0.15 ms^−1^

Site	Subyearling (g)	Yearling (g)	Elevation (m)	Width (m)	% Riffle	% Run	% Pool	% Glide	Depth (cm)	Velocity (ms^−1^)	Substrate (mm)	% Slow and shallow	Cost (Jg^−1^ day^−1^)
LLL	17 (7)	124 (47)	280	5.1 (2.2)	21	57	14	7	22 (12)	0.34 (0.25)	129 (61)	50	212 (24)
LLU	12 (4)	60 (16)	324	5.4 (1.5)	24	24	6	47	21 (11)	0.31 (0.20)	113 (47)	20	206 (26)
LSX	13 (0)	50 (3)	390	4.8 (1.2)	45	35	20	0	20 (15)	0.42 (0.23)	108 (70)	2	197 (27)
MLX	10 (3)	52 (11)	357	3.7 (1.0)	55	20	25	0	17 (12)	0.24 (0.20)	105 (73)	5	217 (23)
ULL	6 (2)	No data	449	4.8 (1.9)	47	6	12	35	13 (8)	0.14 (0.15)	128 (98)	31	201 (13)
ULM	5 (1)	41 (11)	585	4.0 (0.8)	47	24	12	18	10 (8)	0.16 (0.13)	141 (77)	41	198 (26)
ULU	4 (1)	59 (24)	693	3.8 (1.2)	40	40	10	10	12 (8)	0.19 (0.16)	213 (186)	21	190 (21)
UML	8 (4)	77 (8)	411	2.7 (1.2)	15	15	15	55	9 (5)	0.08 (0.09)	119 (66)	36	217 (29)
UMM	5 (2)	25 (4)	472	4.1 (1.2)	95	0	5	0	7 (4)	0.13 (0.10)	124 (96)	48	206 (31)
UMU	4 (1)	29 (19)	629	3.6 (1.0)	32	21	32	16	11 (5)	0.15 (0.14)	238 (226)	17	183 (33)
USL	11 (3)	78 (31)	448	3.0 (1.3)	5	55	15	25	23 (13)	0.41 (0.28)	137 (180)	2	188 (29)
USM	8 (2)	71 (58)	531	4.0 (2.2)	5	30	35	30	31 (16)	0.29 (0.27)	86 (56)	1	179 (27)
USU	5 (1)	58 (29)	575	3.1 (0.7)	20	65	15	0	19 (9)	0.46 (0.30)	107 (73)	1	174 (27)
UWL	4 (2)	54 (47)	438	2.5 (0.8)	23	31	31	15	12 (12)	0.11 (0.11)	85 (48)	83	196 (31)
UWM	4 (1)	36 (26)	490	2.8 (1.0)	63	11	21	5	11 (9)	0.14 (0.13)	103 (58)	22	189 (32)
UWU	3 (1)	37 (26)	525	2.4 (0.7)	47	5	26	21	11 (9)	0.16 (0.15)	159 (98)	26	183 (33)

#### Bioenergetic data

The energetic cost incurred by a given temperature regime was calculated as described in Myrvold and Kennedy (2015a), using data on site-specific temperatures and diets in a bioenergetic model calibrated for juvenile steelhead. HOBO TidbiT v2 temperature loggers (Onset Computer Corporation, Pocasset, MA) installed at each site recorded water temperatures (°C) every 30 min throughout the duration of the study. Daily averages of temperature were used in the subsequent bioenergetic modeling. We used Fish Bioenergetics 3.0 (“Wisconsin model”; Hanson et al. 1997) to calculate the energetic cost incurred by a study site. The model was specified with Thornton and Lessem's (1978) consumption equation, Kitchell et al.'s (1977) respiration equation, Elliott's (1976) waste losses equation, and predator energy density equation number 2 in the package (Hanson et al. 1997) with a predator energy density of 5763 J g^−1^ wet weight (Glova and McInerney 1977). The model was parameterized with the field data on temperature from both 2010 and 2011, and diet data from 431 steelhead over the 2010 season which averaged 4324 J g^−1^ wet weight (Myrvold and Kennedy 2015a). To calculate the ration necessary to maintain body mass for a given period of time under a given temperature regime, we controlled for allometric effects by keeping body mass constant at the start value over that time period (Hewett and Kraft 1993; Myrvold and Kennedy 2015a). We chose to use a 5-g individual for the simulation because it is the average weight of subyearlings in the first months of summer, and we expressed the metabolic cost as the average daily rate from June through October in both 2010 and 2011 (the value of mass is, however, irrelevant for this analysis, as the variation among sites was the primary interest). By holding mass constant, we could compare the maintenance metabolic costs across sites, which were entirely thermally driven patterns. We report metabolic rates as mass-specific rates of consumption (Jg^−1^ day^−1^). Data from June 2011 were limited to the period of June 14–30, because the temperature loggers were washed out by a flood. However, this limitation affected all sites equally and did not affect our analyses. Sample data for average mass, habitat, and bioenergetics are shown in Table 1.

### Statistical analyses

Because we sampled discrete sites across the watershed, we had to account for the clustering in our data when examining the relationship between population density and average mass of the respective age classes. We hence used a linear mixed-effects model, also known as a hierarchical linear model, which can be thought of as a regular regression that allows for clustering of the data points into groups (here, study sites; Raudenbush and Bryk 2002; Littell et al. 2006). Mixed-effects models contain both fixed and random effects and allow for inclusion of higher level covariates, that is, covariates that do not vary across measurements within a group (some readers might refer to this as including sites as random effects). The general form of the self-thinning relationship, 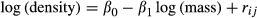 (Yoda et al. 1963), must hence be expanded accordingly to allow for the clustered data structure. If we consider *j* study sites that were chosen at random from a larger population of potential sites, with *i* sampling events in each, the self-thinning relationship can be written as follows:


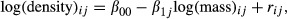
(1)

where *r*_*ij*_∼*N*(0, *σ*^2^). Because the significance of the intercept is unclear (Lobón-Cerviá 2008), we specified all sites as sharing the same intercept, that is, *β*_00_ = *γ*_00_. To allow for site-level variation in the slope *β*_1*j*_, we can rewrite the term to allow for both fixed and random effects. The slope will consist of both the overall (fixed effect) regression slope for all the data and a deviation (random effect) from this overall slope according to site, that is, *β*_1*j*_ = *γ*_10_ + *u*_1*j*_. Here, the overall fixed-effect regression slope is *γ*_10_, and the random effect, or summary of the deviation from this overall slope, is *u*_1*j*_, which is assumed ∼ *N*(0, *τ*_00_). Substituting into equation 1, we obtain the following expression for the self-thinning relationship, which will be referred to as the base relationship:


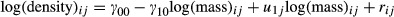
(2)

To partition the variance among the two levels, that is, the sampling event level and the site level, the variance contributed by each level toward the total variance of density data was examined. We hence computed an unconditional, or empty, model for each age class and recorded the variation in each level (Raudenbush and Bryk 2002).

The second objective of the study was to explain the potential causes of the variation in the relationship between mass and density that occurs among different study sites. We therefore included a set of site-level predictors that described various habitat conditions that can be important in determining the slope of the relationship. These habitat factors were included as *cross-level* interaction terms (Raudenbush and Bryk 2002). A cross-level interaction affects the mass–density relationship according to the value of that predictor, which varies by study site. The site-level predictor, denoted pred, was hence included in the slope term as follows: *β*_1*j*_ = *γ*_10_ + *γ*_11_ pred _*j*_ + *u*_1*j*_. Substituting this into equation 1, we obtain the following relationship between mass and density at sampling occasion *i* in site *j*, with a site-level habitat factor pred:


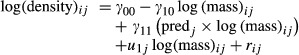
(3)

The gammas in this equation refer to the fixed effects, which are equivalent to betas in a regular regression, whereas the *u* refers to the random effect of site. Finally, the *r* refers to the residual of the relationship, as in a normal regression. The model structure in equation 3 was used to test hypotheses of which habitat factors could explain the site-to-site variation in thinning slopes according to Table 2, where the respective factors would take the place of the *pred* term above.

**Table 2:** Candidate models for explaining the site-level variation in self-thinning slopes. Shown for the respective age classes is the AIC value for the model that included the site-level covariate *pred* in the following equation 3

Variable *pred* in equation 3	AIC subyearling	AIC yearling
Energetic cost (Jg^−1^ day^−1^)	174.7	111.2
Riffle habitat (%)	171.0	131.3
Run habitat (%)	171.9	131.3
Pool habitat (%)	174.8	128.0
Glide habitat (%)	175.3	131.3
Average water depth (cm)	168.5	130.9
Average flow velocity (ms^−1^)	170.0	131.0
Average substrate size (mm)	172.4	129.0
Slow and shallow (%)	175.2	131.0

All analyses were conducted using PROC MIXED in SAS 9.2 (SAS Institute Inc., Cary, NC), with maximum-likelihood as the estimator, the Kenward and Roger (1997) approximation of degrees of freedom, and an unstructured covariance matrix (Littell et al. 2006). The unstructured covariance structure was deemed the most appropriate for the data because it accounts for the correlated error structure among observations within a study site while not constraining their values. We used an information-theoretic criterion to find the best approximating model of the data (Akaike 1973; Burnham and Anderson 2002). Akaike's information criterion (AIC) is given as follows:


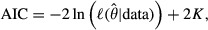


where 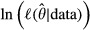 is the maximum log-likelihood for a model, and *K* is the number of estimable parameters in that model. The model for which AIC is minimal is selected as best for the given data. The models were ranked using the simple AIC differences, *Δ*_*i*_, given as follows: *Δ*_*i*_ = AIC_*i*_ *−* AIC_min_. The AIC values of the models were normalized so that they sum to 1 to make interpretation and inference easier, and we report on the Akaike weights of the models examined. We only provided parameter estimates for those models in each set which obtained substantial relative support (i.e., AIC_*i*_ *−* AIC_min_ < 2). We examined the residual plots to make sure the error structures were normally distributed with means of zero and constant variances.

## Results

### Prevalence and strength of self-thinning curves

**Figure 3:**
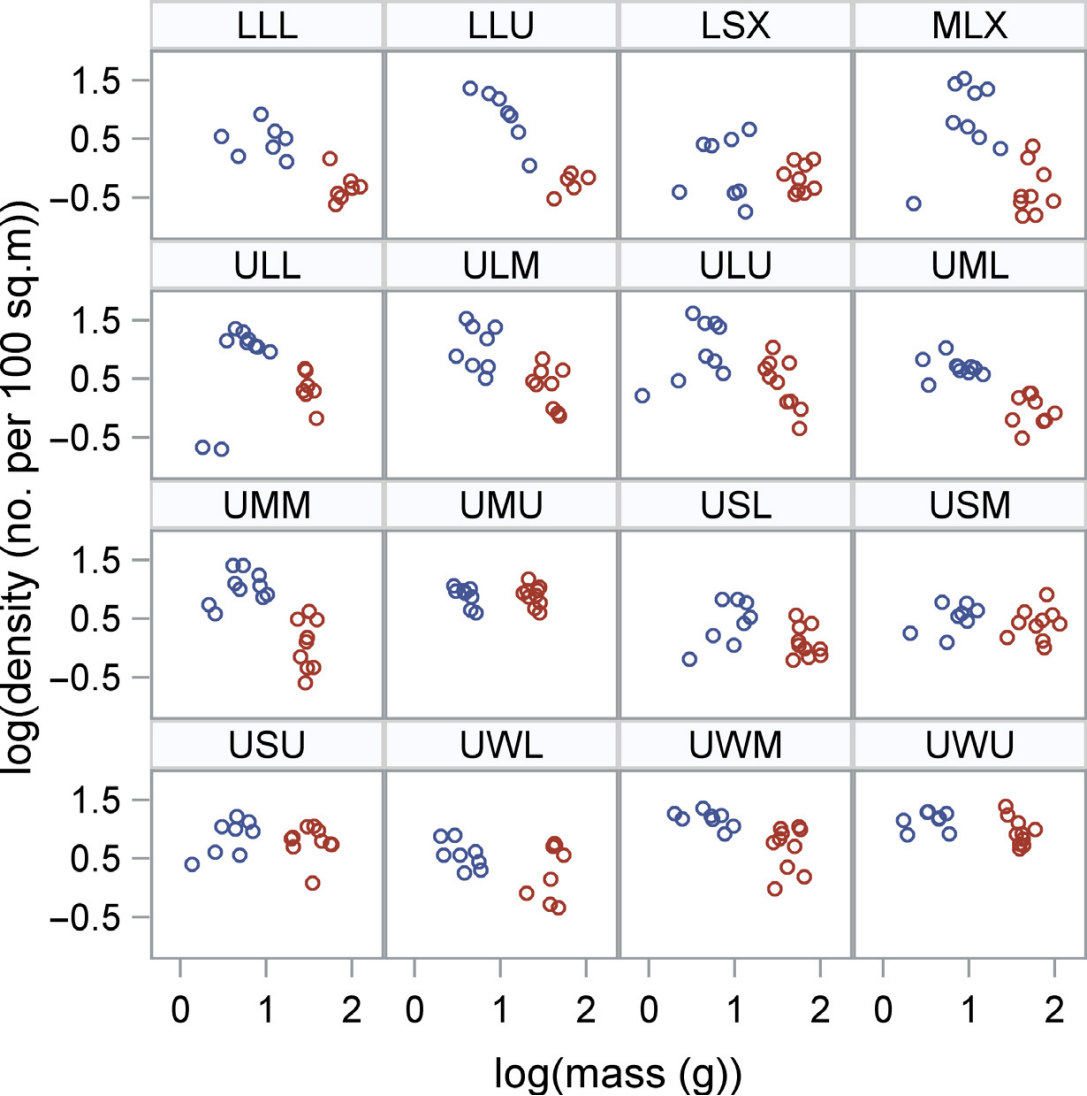
The relationship between average mass and density for subyearling (blue circles) and yearling (red circles) steelhead in 2010 and 2011 across the 16 study sites in the Lapwai Creek watershed, USA. The data are based on an average of five visits to each site each year and are shown on a logarithmic scale with base 10.

We were able to obtain 143 estimates of subyearling steelhead densities and 135 estimates of yearling densities. Densities varied considerably within and among study sites (Fig. 3). Densities of subyearling steelhead were higher than yearling densities in most study sites. Average yearling densities were lower than 10 fish per 100 m^2^, and subyearling densities were lower than 20 fish per 100 m^2^ in most cases.

When we analyzed the overall relationship between average mass and population density while accounting for site-level variation, we found a significant negative relationship for yearling steelhead, with a slope of −0.529 (SE = 0.221). No significant self-thinning was found in subyearling steelhead, which had a slope of 0.269 (SE = 0.18; *see* base models in Table 3). The estimates for each site are given in Table 4, which show a higher proportion of yearling slopes being negative and several subyearling slopes being positive.

**Table 3:** Parameter estimates for the empty (unconditional) model, the base model, and the best approximating models from the model selection of site-level factors which could explain the variation in thinning slopes. Shown are the model structures, parameter estimates with standard error in parentheses for the variables, and the intraclass correlation for site-level factors, with the proportion of explainable variation that was explained by the inclusion of the site-level variable in parentheses

Age class and type	Model	Variable	Estimate (SE)	ICC (% variance explained)
Subyearling				
Empty	log(density)_*ij*_ = *γ*_00_ + *u*_1*j*_ + *r*_*ij*_	Intercept *γ*_00_	0.77 (0.077)	
		Variance *τ*_00_	0.076 (0.034)	31
		Residual *σ*^2^	0.17 (0.022)	
Base	log(density)_*ij*_ = *γ*_00_ + *γ*_10_ log(mass)_*ij*_ + *u*_1*j*_ log(mass)_*ij*_ + *r*_*ij*_	Intercept *γ*_00_	0.589 (0.119)	
		Slope *γ*_10_	0.269 (0.180)	
		Variance *τ*_00_	0.132 (0.060)	45
		Residual *σ*^2^	0.164 (0.0213)	
Depth	log(density)_*ij*_ = *γ*_00_ + *γ*_10_ log(mass)_*ij*_ + *γ*_11_ depth_*j*_ · log(mass)_*ij*_ + *u*_1*j*_ log(mass)_*ij*_ + *r*_*ij*_	Intercept *γ*_00_	0.563 (0.119)	
		Slope *γ*_10_	0.891 (0.286)	
		Cross-level *γ*_11_	−0.0377 (0.0130)	
		Variance *τ*_00_	0.0755 (0.0386)	32 (1)
		Residual *σ*^2^	0.164 (0.0212)	
Velocity	log(density)_*ij*_ = *γ*_00_ + *γ*_10_ log(mass)_*ij*_ + *γ*_11_ velocity_*j*_ · log(mass)_*ij*_ + *u*_1*j*_ log(mass)_*ij*_ + *r*_*ij*_	Intercept *γ*_00_	0.577 (0.119)	
		Slope *γ*_10_	0.721 (0.257)	
		Cross-level *γ*_11_	−1.87 (0.727)	
		Variance *τ*_00_	0.0791 (0.0423)	32 (4)
		Residual *σ*^2^	0.165 (0.0216)	
Yearling				
Empty	log(density)_*ij*_ = *γ*_00_ + *u*_1*j*_ + *r*_*ij*_	Intercept *γ*_00_	0.261 (0.103)	
		Variance *τ*_00_	0.159 (0.0603)	61
		Residual *σ*^2^	0.103 (0.0129)	
Base	log(density)_*ij*_ = *γ*_00_ + *γ*_10_ log(mass)_*ij*_ + *u*_1*j*_ log(mass)_*ij*_ + *r*_*ij*_	Intercept *γ*_00_	1.15 (0.351)	
		Slope *γ*_10_	−0.529 (0.221)	
		Variance *τ*_00_	0.0445 (0.0179)	30
		Residual *σ*^2^	0.105 (0.0132)	
Cost	log(density)_*ij*_ = *γ*_00_ + *γ*_10_ log(mass)_*ij*_ + *γ*_11_ cost_*j*_ · log(mass)_*ij*_ + *u*_1*j*_ log(mass)_*ij*_ + *r*_*ij*_	Intercept *γ*_00_	1.29 (0.319)	
		Slope *γ*_10_	2.17 (0.525)	
		Cross-level *γ*_11_	−0.0142 (0.0023)	
		Variance *τ*_00_	0.00920 (0.00503)	8 (94)
		Residual *σ*^2^	0.105 (0.0132)	

**Table 4:** Deviations from the overall mass–density relationship for each site and age class. For each study site, the table shows the local deviation from the overall thinning slope (the fixed-effects estimate) and its standard error, the point estimate for the site-specific slope, the degrees of freedom, the *t*-value, and the associated *P*-value

	Yearlings					Subyearlings				
Site	Deviation from *γ*_*10*_ (SE)	Estimated slope	df	*t*-value	*P*(>¦*t*¦)	Deviation from *γ*_*10*_ (SE)	Estimated slope	df	*t*-value	*P*(>¦*t*¦)
LLL	−0.221 (0.0849)	−0.750	52	−2.61	0.011	−0.332 (0.172)	−0.063	52	−1.93	0.059
LLU	−0.205 (0.0919)	−0.734	59	−2.23	0.030	−0.041 (0.168)	0.228	51	−0.25	0.810
LSX	−0.196 (0.0796)	−0.725	47	−2.46	0.018	−0.807 (0.166)	−0.538	51	−4.86	<0.0001
MLX	−0.311 (0.0797)	−0.840	47	−3.9	0.0003	0.027 (0.161)	0.296	49	0.17	0.87
ULL	−0.017 (0.0936)	−0.546	61	−0.18	0.86	0.152 (0.183)	0.421	54	0.83	0.41
ULM	0.025 (0.0819)	−0.504	50	0.31	0.75	0.246 (0.193)	0.515	54	1.28	0.21
ULU	0.033 (0.0818)	−0.497	50	0.4	0.69	0.340 (0.203)	0.609	53	1.68	0.10
UML	−0.142 (0.0797)	−0.671	47	−1.78	0.081	−0.143 (0.165)	0.126	51	−0.87	0.39
UMM	−0.188 (0.0885)	−0.717	57	−2.13	0.038	0.265 (0.180)	0.534	54	1.48	0.15
UMU	0.305 (0.0936)	−0.224	59	3.26	0.0019	0.134 (0.223)	0.403	49	0.6	0.55
USL	−0.045 (0.0780)	−0.574	44	−0.58	0.56	−0.320 (0.168)	−0.051	51	−1.91	0.062
USM	0.117 (0.0780)	−0.412	45	1.5	0.14	−0.260 (0.179)	0.009	54	−1.45	0.15
USU	0.265 (0.0828)	−0.264	51	3.21	0.0023	0.178 (0.218)	0.447	50	0.82	0.42
UWL	−0.010 (0.0850)	−0.539	54	−0.11	0.91	−0.258 (0.228)	0.011	48	−1.13	0.26
UWM	0.223 (0.0796)	−0.306	48	2.8	0.0074	0.365 (0.198)	0.634	54	1.85	0.070
UWU	0.367 (0.0808)	−0.162	49	4.54	<0.0001	0.456 (0.229)	0.725	47	1.99	0.052

**Figure 4:**
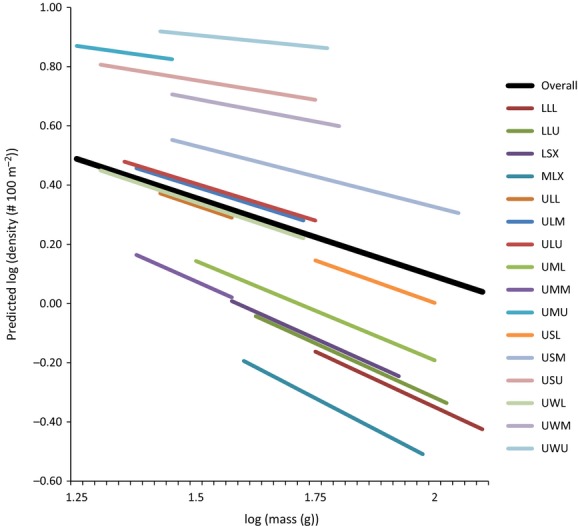
Predicted self-thinning curves by study site for yearling steelhead in the Lapwai Creek watershed, USA. Shown in bold is the overall weighted thinning curve for all sites combined.

The reason we found a strong negative relationship in yearling steelhead, but not in subyearlings, could owe to a larger within-site spread in the densities of subyearlings and consequently more overlap among study sites (Fig. 3). The intraclass correlation (which is a measure of the proportion of the total variance in the density data that can be attributed to site-level factors) was 61% in yearlings, nearly twice that of subyearling steelhead (31%; *see* empty model in Table 3). The densities of yearling steelhead were hence less variable within a site over the course of the study, and there were more discernible density differences among the sites. A plot of the predicted thinning slopes for yearling steelhead is given in Fig. 4.

### Habitat and bioenergetic effects explaining site-level variation

To explain the variation in thinning slopes among the 16 study sites, we included factors that describe certain habitat conditions that vary across the Lapwai Creek watershed. These factors were included as a cross-level interaction with the average mass at the sampling event (equation 3), hence directly imposing a site-level constraint on the mass–density relationship. A *steeper* slope means that the site-specific regression line falls below the weighted average for the entire study system.

We found that energetic cost best explained the variation in thinning slopes among sites for yearling steelhead (Table 2). There was no other model within 2 AIC points of this model, and it received 99% of the relative support among the models considered. The factor greatly reduced the intraclass correlation, which means that a large proportion of the explainable variation at the site level was explained by this factor (Table 3). In other words, sites in which fish expended more energy on basal metabolism, as a result of warmer temperatures, exhibited a steeper self-thinning slope.

For subyearling steelhead, average water depth (w = 45%) and flow velocity (w = 21%) best described the habitat conditions that cause variation in thinning slopes among sites (Table 2). These two models collectively received 66% of the relative support among the competing models. Taken together, these two models show that subyearling thinning slopes were steeper in sites with greater water depth and faster flow velocity.

## Discussion

We found that self-thinning in juvenile steelhead cohorts was not ubiquitous across all study sites. In general, self-thinning slopes were shallower than predicted by theory, and local habitat conditions had significant mediating effects on the slope of the thinning curve. First, only yearling steelhead exhibited a significant self-thinning relationship, but the slope (−0.53) was shallower than predicted by the territory space (−0.86; Grant and Kramer 1990), food consumption (−0.73; Steingrimsson and Grant 1999), and metabolic rate (−0.87; Steingrimsson and Grant 1999) hypotheses. Second, specific abiotic factors explained a relatively large portion of the variation in the strength of the self-thinning among the study sites. For subyearling steelhead, self-thinning slopes were steeper in sites with greater water depth and faster flow velocity, whereas slopes in yearlings were steeper in sites that incurred a greater metabolic cost.

Identifying the factors by which a population is limited is an important step in any wildlife management, particularly in threatened populations where mitigation and restoration efforts are undertaken (Sibly and Hone 2002; Armstrong 2005). Self-thinning can be used to diagnose density dependence in a population on time spans shorter than its life cycle and estimate carrying capacity for the given size distribution (Yoda et al. 1963; Westoby 1981). However, the exact mechanisms causing these patterns are impossible to discern in observational studies (Dunham and Vinyard 1997) and difficult even in experimental studies (Keeley 2003). Several studies have attempted to identify the underlying mechanism causing self-thinning patterns in stream salmonids. Two major hypotheses exist to explain how and why population density scales with average body mass in populations. The food hypothesis focuses on the allometry of metabolism or food consumption (Bohlin et al. 1994; Steingrimsson and Grant 1999). The space hypothesis concerns the territorial nature of stream salmonids in that the area of their territories increases with fish mass (Grant and Kramer 1990), analogous to shading in plants (Lonsdale and Watkinson 1983). Although several studies have observed the thinning pattern (e.g., Elliott 1993; Rincón and Lobón-Cerviá 2002; Lobón-Cerviá 2008), few studies have actually tested these hypotheses. Keeley (2003) tested the predictions from the food and space hypotheses in an experimental cohort of juvenile steelhead and found stronger support for the food consumption hypothesis, which predicts a thinning slope of 0.73. However, the confidence intervals overlapped the values predicted from metabolic rate and territory size, and it consequently was impossible to reject those hypotheses. The similarity in theoretical predictions thus makes them difficult to distinguish even with well-designed experiments (Dunham and Vinyard 1997; Keeley 2003).

The finding of no apparent thinning in subyearling steelhead, but significant thinning in yearling cohorts, bears relevance to the consideration of age structuring in juvenile salmonids. Our results likely owe to recruitment limitation and/or stage-dependent habitat limitation (Lobón-Cerviá 2008). First, the steelhead populations might be limited by recruitment, or by survival during the first free-swimming phase before we could enumerate the cohort. As a result, it would be possible that the cohort does not reach the carrying capacity of the habitat until achieving a larger body size, with the consequence that the thinning slopes were shallower due to delayed onset of density dependence. Such “segmented” density dependence has been demonstrated in brown trout in Spain (Rincón and Lobón-Cerviá 2002; Lobón-Cerviá 2008) and leads to a shallow relationship between mass and density when the average mass is low, followed by a steeper slope after a threshold mass is reached. Above this threshold, the stream section cannot sustain further increases in average mass without a proportional decrease in density.

Second, the limitation by certain characteristics of the habitat can be more pronounced as individuals grow in size and require more resources. Studies have shown how environmental conditions can determine population size by limiting survival or recruitment (Hayes et al. 1996; Jensen and Johnsen 1999; Armstrong 2005; Einum and Nislow 2005), and a few have linked variation in the strength of density dependence directly to habitat characteristics (Steingrimsson and Grant 1999; Lobón-Cerviá 2008). Salmonid fry tend to prefer slower-flowing microhabitats when compared to preferences of older conspecifics (Bjornn and Reiser 1991; Beecher et al. 1993; Quinn 2005), and the thinning slopes showed a clear negative relationship to both depth and velocity in our study. A previous study in the Lapwai Creek watershed found that the negative effects on individual growth rates resulting from high temperatures and food limitation (termed energetic bottlenecks) increased with body size in juvenile steelhead (Myrvold and Kennedy 2015a). This could explain the pattern that yearling steelhead thin more consistently overall and more so in sites where a larger energetic cost is incurred. Growth opportunities and densities of salmonids typically vary spatially within river networks, and it is hence likely that local differences in ambient habitat conditions can produce a corresponding range of mass–density relationships (Gibson 2002; Ebersole et al. 2006; Myrvold and Kennedy 2015b).

Determining the extent to which self-thinning in mobile animals occurs in natural systems is complicated by individual movement. Because our study design focused on revisits to established study sites, we could not separate mortality from movement out of the study sites. Both mortality (Rose et al. 2001; Hartson and Kennedy 2015) and movement and migration rates (Einum et al. 2012) are known to be density dependent. Further, mobile animals can move in relation to ambient habitat conditions and ontogenetic changes (Armstrong 2005; Einum et al. 2006; Satterthwaite et al. 2009), which would be reflected in local densities and subsequently in the site-specific thinning curves. Movement in summer is largely nonmigratory in the Lapwai system, where most fish out-migrate during peak spring runoff (NMFS 2010, Hartson & Kennedy 2015). Our sampling approach would therefore likely capture the variability in the nonmigratory movement in relation to the habitat factors the individuals were selecting for and not be confounded by migration.

Finally, we note that stochastic events and density-independent factors can directly limit population size and obscure density-dependent processes (Begon et al. 1996; Jensen and Johnsen 1999; Myrvold and Kennedy 2015b). Because density-independent factors can cause mortality and movement, we would expect a negative population trend over time, in the absence of density dependence. At the same time, juveniles gain mass over their first months and years of life, which makes mass positively correlated with time and density negatively correlated with time. The thinning slope, however, shows the relationship between average mass and density and represents a mechanism rather than a trend. We are therefore confident that the thinning slopes found in this study represent density dependence as described in the literature (e.g., Dunham and Vinyard 1997; Steingrimsson and Grant 1999; Rose et al. 2001; Lobón-Cerviá 2008).

In conclusion, our results show that density dependence can be more pronounced during certain stages of a species' life history and that environmental factors can affect the extent to which density dependence is manifested. In natural systems, it is not surprising that the strength of density dependence is dynamic or spatially variable (Lobón-Cerviá 2008) because of local variation in population densities, habitat productivity, and habitat suitability. Our results highlight the role of the physical environment in mediating these density-dependent effects and that they can pose differential effects on different life stages in stream salmonids. In light of the higher temperatures predicted for streams in the western United States with a warming climate (Barnett et al. 2005), our results indicate that the capacity of natal streams to support rearing salmonids could decrease in the absence of a proportional increase in food resources.
